# Broadband compact rectenna system using a Wilkinson power divider to harvest microwave energy

**DOI:** 10.1038/s41598-025-02555-1

**Published:** 2025-05-21

**Authors:** Milad Nosrati, Pejman Rezaei, Mohammad Danaie, Jafar Khalilpour

**Affiliations:** 1 Electrical and Computer Engineering Faculty, Semnan University, Semnan, Iran; 2 Faculty of Electrical Engineering, Tarbiat Modares University, Tehran, Iran

**Keywords:** Engineering, Electrical and electronic engineering

## Abstract

In the present study, a broadband symmetrical rectifier high-efficiency low-complexity single-layer cost-efficient system at 2–18 GHz frequency with horizontal and vertical polarization in receiver antennas has been proposed for harvesting radio frequency energy. The rectenna system circuit consists of two ridged horn antennas with separate linear horizontal and vertical polarization that receive the RF waves. Then, a uniform and integrated rectifier circuit is presented using two power dividers and a Wilkinson combiner to divide and combine the desired power in the system. The rectifier part uses a commercial Schottky diode (HSMS-2850). Using this structure on the output of the rectifier circuit, the vertical and horizontal polarization, as well as a combination of the two, are provided with high power transformation efficiency and the lowest power drop (equally 3 dB) to harvest various types of RF energy. The new rectifier circuit consists of a unique structure and a cheap and available substrate (RO4003) all over the frequency range of 2–18 GHz. The designed prototype has an average power conversion efficiency of 32% for very low input power, around − 5 dBm. In addition, this rectifier circuit has a voltage of 3.55 V at the power of -5 dBm. Considering the simultaneous use of two receivers, when the relative phase of input signals ranges from 0 to 360 degrees, an efficiency of 35 to 40% can be observed.

## Introduction

**Fig. 1:**
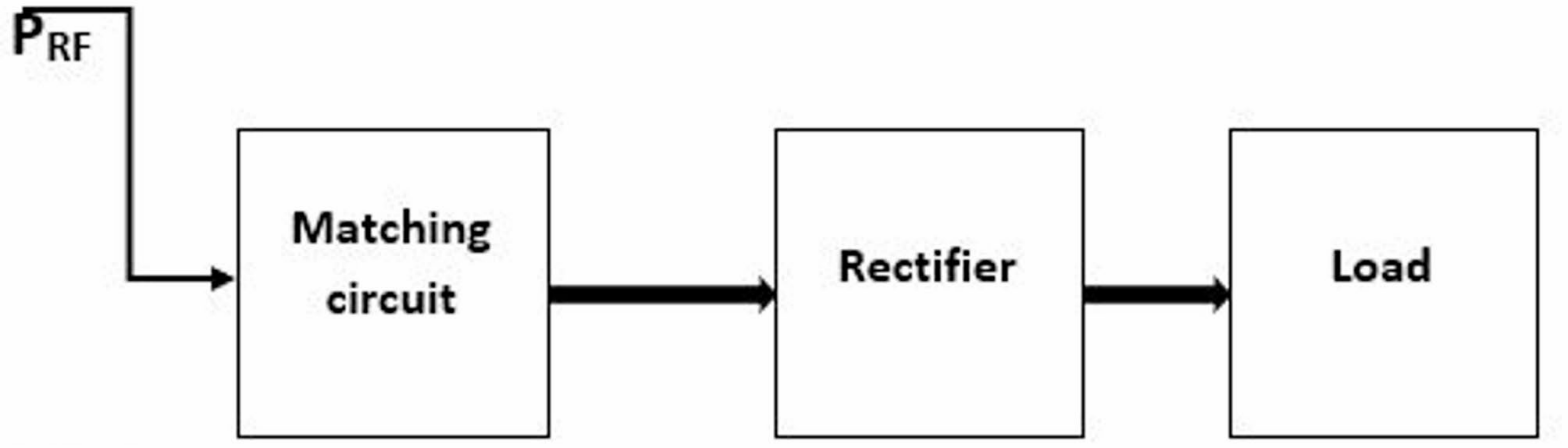
Common structure of rectenna systems in the harvesting of environmental radio energy.

Recently, the use of antennas beside various parts of a system has become very popular among telecommunications engineers. Attention to the rectenna system and ways to improve its basic parameters has been very impressive^1–4^. These antennas can be used in the satellite communication system in astronomy and industrial sciences^5,6^, in modern medical science, in various types of radar systems, and in microwave energy rectifier circuits (rectennas)^7–11^. Energy harvesting is one of the important subjects in today’s green economy. The use of energy emitted from numerous devices in Radio Frequency (RF) is one of the various aspects of energy harvesting in the present time and future. On the other hand, the use of RF sources is one of the possible approaches for transmitting and harvesting wireless power^12,13^. A rectenna is one of the common devices for energy harvesting, which is a circuit consisting of an antenna, a matching circuit, a rectifier, and a low-pass filter. A rectenna is an antenna used to harvest wireless power and convert it into DC power. A common rectenna system consisting of an antenna, matching circuit, rectifier, and load is presented in Fig. 1.

**Fig. 2:**
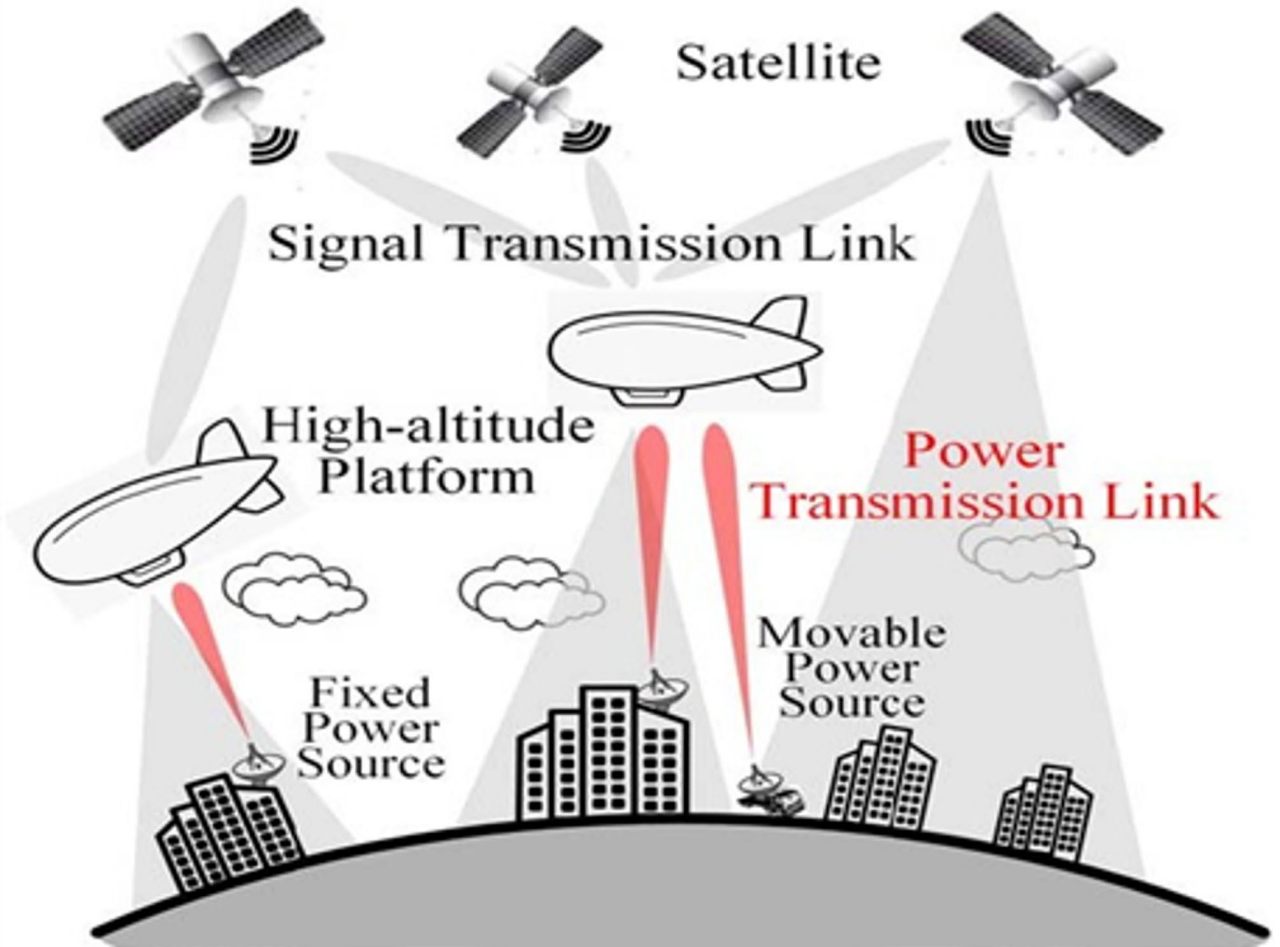
A schematic of the architecture of an integrated space-air-ground network.

Rectenna is increasingly progressing in all frequency bands as well as the high-volume, cost-efficient integrated space-air-ground networks of the 6G world converged communication systems. Using ground sources, microwave wireless power transmission provides potential solutions to charge different aerial platforms, such as airships, unmanned aerial vehicles (UAVs), and high-altitude platform stations, to support true global wireless communications anywhere, anytime. Figure 2 shows an integrated spatial-aerial-ground network of a 3-D network taken by satellites (e.g., Star Link) and high-altitude platform stations. Through a large-scale, highly directional beam and rectenna array, microwave wireless power transmission can reach a high efficiency and receive RF energy. Therefore, rectenna systems have various applications in today’s telecommunication, and they will be increasingly used in the future.

This system is not merely used to supply voltage to various devices. It can be used for multiple purposes regarding the end of the fossil fuel era in the near future. Microstrip antennas^14^, dipole antennas^15–17^, monopole antennas^18,19^, slot antennas^20–22^, horn and triangular patch antennas^23,24^ is commonly used in this system. The matching circuit is another part of the rectenna system that is responsible for the adjustment of the impedance between the receiver antenna and rectifier. This part has been excluded from some studies to compress the rectifier circuit^25^. In some rectenna systems, also, the matching circuit or the mid-pass filter^26,27^. Also, in some studies, compressed mid-pass filters (nanodisk resonators) have been used in designs for high frequencies^28^. In all studies on the rectennas, mostly, one specific frequency, or ultimately, two^29–32^, three^33–35^, or four^36,37^ frequency bands have been evaluated (also, in some articles, more than four bands have been researched in a limited way)^38–40^. However, in most studies evaluating more than two bands, all the fundamental parameters in the rectifier antenna are not normal and optimal. In the rectifier antennas, multiple forms are used to divide the power and energy unequally, to be used for various applications^41–43^. Also, in some studies, some circuits that automatically polarize in the rectifier are used^44,45^.

It should be noted that in these circuits, the Ku band has been used in lower frequencies. Recently, regarding the various applications of the rectenna systems, they have been abundantly evaluated for high frequencies^46–48^. However, they have not been studied for low frequencies. Energy harvesting in the rectenna systems is progressing due to the consideration of clean energy in all industries and advanced countries in the fields of wireless power transmission and microwave energy harvesting in the field of self-charging batteries and clean and low-cost energy^49–51^. On the other hand, in addition to research on very low input power levels, when the relative phase of the input signals changes over a wide range, variations in power conversion efficiency are investigated over a broad range^52–54^. The research in the field of microwave rectifiers is advancing rapidly, aiming not only to enhance sensitivity and power conversion efficiency (PCE) but also to expand their operational parameters, including bandwidth, input power range, and load conditions^54–57^.

. In electronic circuits and systems, the problem of power transmission (division and combination) is one of the most important parameters that determine the circuit performance and telecommunication system features. The minimum three-port microwave circuit elements that divide the input power equally or unequally to the number of output branches are called power dividers. In the present study, a broadband circuit that simultaneously harvests the ambient power of the signals in the environment is presented.

However, in this field, there are still defects and problems that need improvement and progress in receiving radio frequency energy. In the present study, a receiver antenna that covers frequencies from 1 GHz to the Ku band has been used. Also, in the next part, an integrated rectifier circuit with a commercial Schottky diode and a conventional and cheap board was used. One of the most important parts of the present study is the use of a Wilkinson power divider and combiner at the frequency of 1–18 GHz (equally 3 dB). The most important feature of the present study is dealing with and considering all fundamental parameters of the rectenna system, including the bandwidth frequency (1–18 GHz), the simultaneous use of two different antennas with horizontal and vertical polarizations, and a combination of the two on the output circuit, system efficiency in very low power density, appropriate transformation efficiency through the entire frequency band, and a suitable voltage on the output circuit of the rectifier.

## Design principles and rectenna setup

Recently, in studies on rectenna systems, a flowchart of the design process has been provided to give readers a broader perspective. In this example of flowcharts^55^, potential issues and challenges in each section are broadly identified, making it a reasonable graphical and descriptive approach. In the suggested rectenna arrangement, the various components of the rectenna, including the horn receiving antenna, the Wilkinson power divider, the rectifier section, and the load resistor, are individually designed. Subsequently, an integrated rectenna system comprising all these components is designed for harvesting radio frequency energy.

**Fig. 3:**
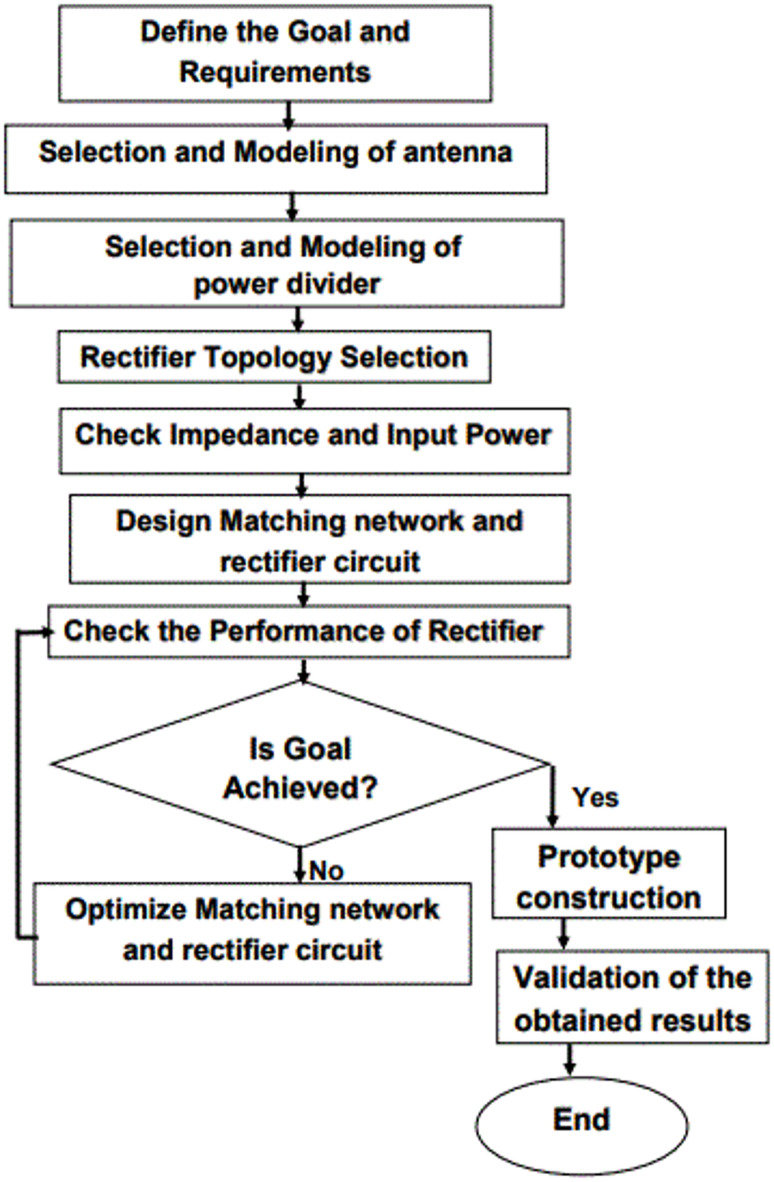
Flowchart of the proposed ultra-wideband rectenna design.

For rectification, a Schottky diode is used in the rectifying circuit. Therefore, understanding the diode’s impedance behavior is essential to designing a proper matching circuit. Therefore, the main goal is to determine the equivalent circuit model of the diode and then, using small-signal analysis, the impedance behavior of the chosen diode. Figure 3 shows the step-by-step process for designing an effective rectifier.

First, the Horn receiving antenna, as the initial component of the rectenna system, is designed and simulated using HFSS and CST high-frequency software. If the antenna’s return loss is appropriate across the entire frequency range, the receiving antenna is fabricated. In the second part, a new compact broadband Wilkinson power divider is designed and simulated. If the appropriate coupling factor is achieved, the power divider is fabricated. The next section involves the model and design of the rectifier circuit using a suitable Schottky diode in compact form. This section directly impacts the overall performance and efficiency of the circuit, thus requiring careful attention in the design of its elements. In the subsequent part, different load resistors are used to check the conversion efficiency, output power, and other essential parameters of the rectenna. If all components are optimized, the proposed broadband rectenna can be fabricated.

### Proposed system receiver antenna

The main antenna used in this design is the horn antenna. These antennas are among the ultra-wideband antennas that work at the frequency range of 2–18 GHz and have a bandwidth of 16 GHz. These antennas have a half-power beam width at the horizontal direction of 90 degrees, and a vertical direction of 60 degrees. It should be noted that all specifications mentioned for the first category should apply to vertical and horizontal orthogonal polarization. In the following, a schematic of the antennas and the separate antennas designed in the present study are presented. Then, the behavior of the proposed antennas in the desired frequency bands will be discussed.

### Antenna with a polarization of two vertical blades

Horn antennas are particularly well suited for rectenna systems operating in the 2–18 GHz frequency range due to their wide bandwidth, high gain, low loss, ability to handle high power levels, and precise directional capabilities. Their robustness and well-defined radiation pattern make them ideal for efficiently harvesting energy in this frequency range. These features enable horn antennas to offer high efficiency in rectenna applications, making them highly effective in harvesting energy from weak or distant RF sources in complex real-world environments.

**Fig. 4:**
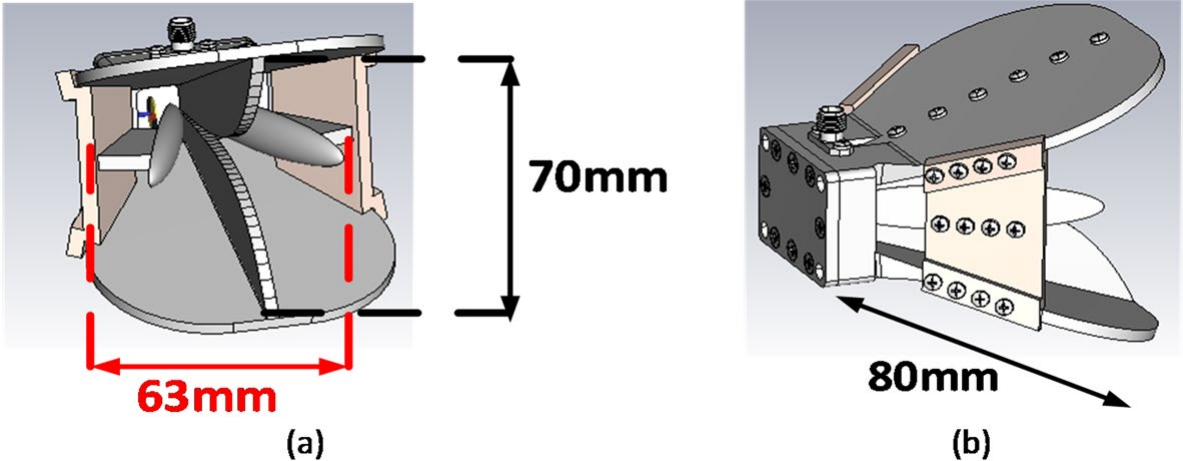
View of the antenna with vertical polarization in the frequency range of 2–18 GHz (a) General view of the antenna in the 3D software ANSYS HFSS, and (b) General view of the antenna including the packaging and all components required for manufacturing and machining in SOLIDWORKS software.

We have to separately evaluate the vertical and horizontal antennas first. To make the antenna wideband, ellipsoids are used close to the antenna blades. It makes the antenna wideband and compresses its size. A schematic of the antenna in the vertical mode is presented in Fig. 3. As shown in Fig. 4, the size of the antenna is 63 × 70 × 80 mm. It is worth noting that Fig. 4a shows the antenna with vertical polarization in the frequency range of 2–18 GHz, presenting the general view of the antenna in the 3D software ANSYS HFSS with version number ANSYS HFSS 2022 R1^58^. Additionally, Fig. 4b shows the general view of the antenna, including the packaging and all components required for manufacturing and machining in SOLIDWORKS software with version number SOLIDWORKS 2023 SP2.0 (Service Pack 2.0)^59^.

As was mentioned before, ellipsoids are used near the blade to increase the beam width in all frequency bands of 2–18 GHz. As shown in this figure, an SMA connector is used for antenna feeding. The connector feeder is connected to one of the blades through a metal pin, and the connector’s body also directly touches the horn antenna body. To fabricate the antenna, the ellipsoids are fabricated separately and connected to the antenna body through a PTFE wall.

**Fig. 5:**
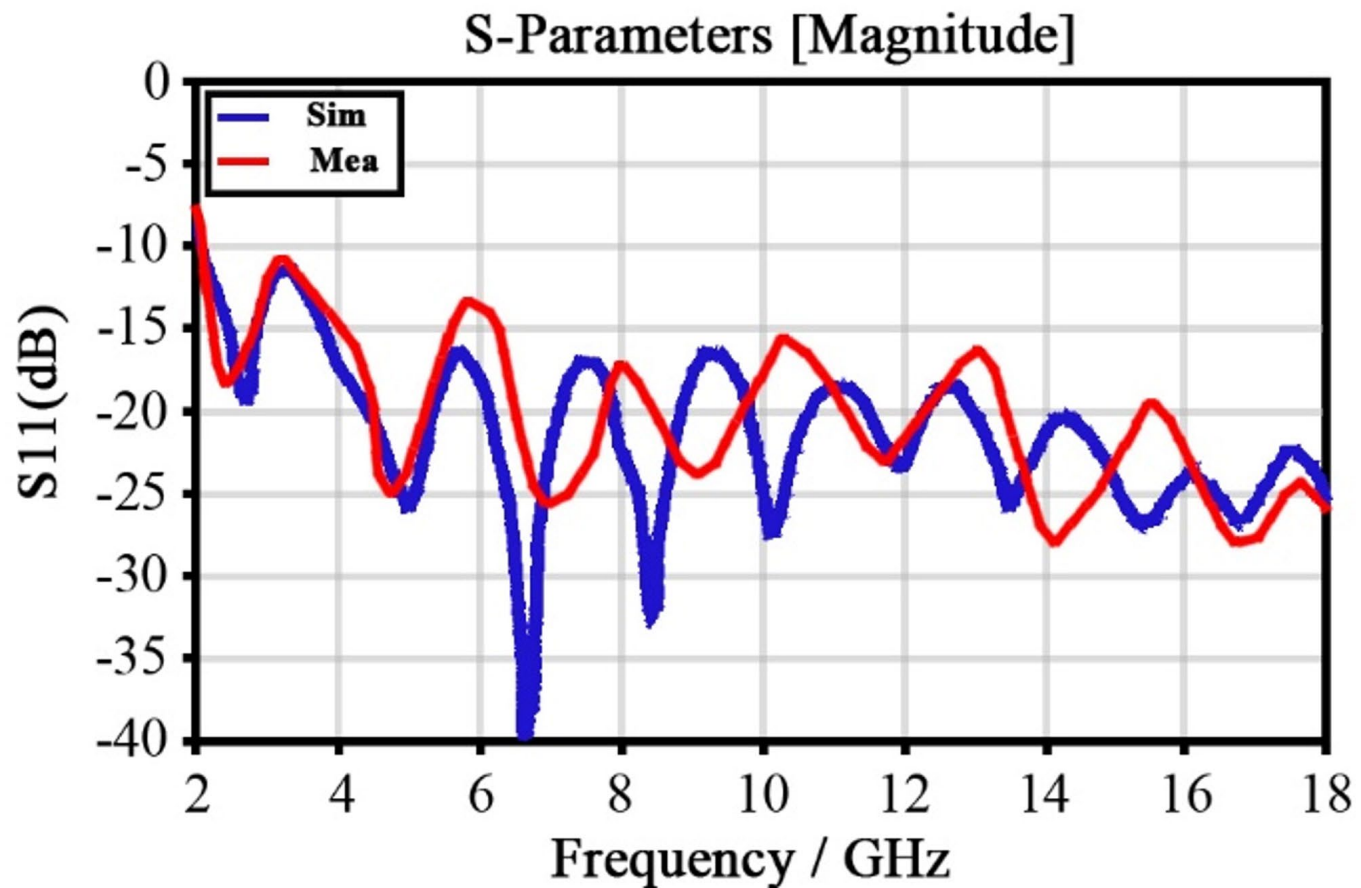
The diagram of the return loss double blade horn antenna with vertical polarization.

**Fig. 6:**
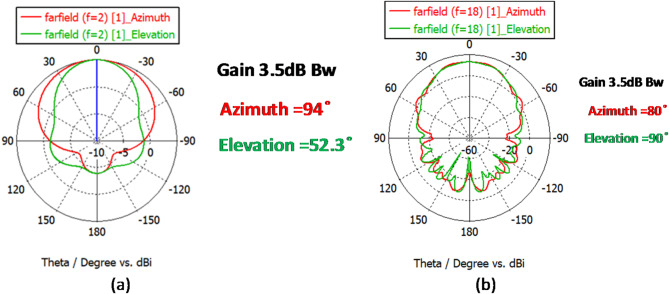
The radiation pattern of a two-blade horn antenna with vertical polarization at (a) 2 GHz frequency and (b) 18 GHz frequency.

Figure 5 shows the return loss graph. It can be observed that the antenna works in the 2–18 GHz range. As seen in Fig. 6, considering the need to receive radiation in all directions, the radiation pattern has a gain value of 7 dB and a half-power beam width in the horizontal and vertical directions of 90 and 60 degrees, respectively. In other words, the gain value at the edge of the half-power beam width is equal to 3.5 dB. Regarding the unique structure of the antenna that works as both a receiver and transmitter, the non-orthogonal polarization also did not confine the orthogonal polarization of the receiver/transmitter antennas.

### Antenna with the polarization of two horizontal blades

**Fig. 7:**
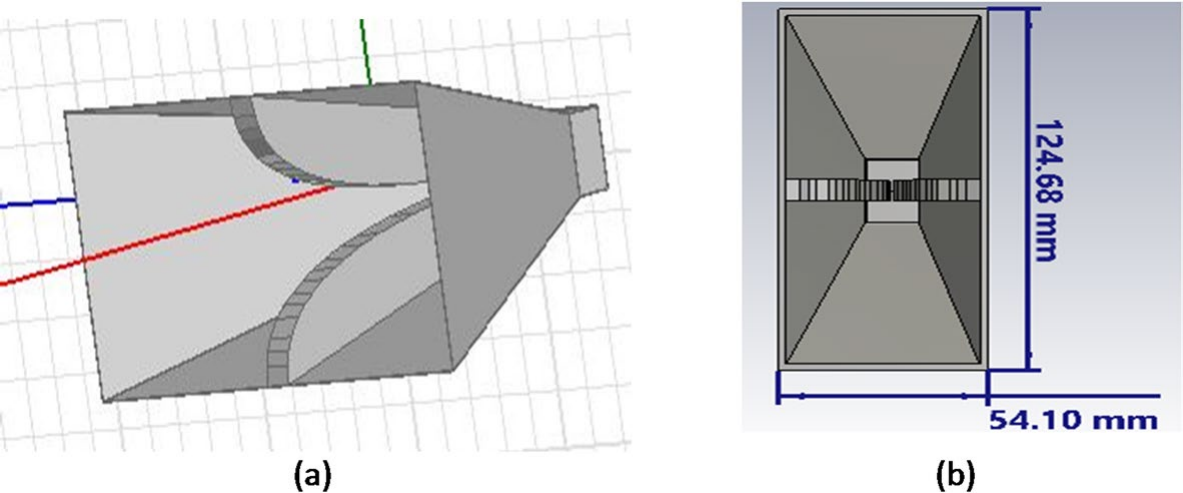
An example of a horizontally polarized horn antenna operating in the 2–18 GHz frequency band. (a) General view of the antenna in the 3D software ANSYS HFSS. (b) Side view of the antenna in SOLIDWORKS software.

In this section, the radiation pattern of a double-ridged horn antenna with horizontal polarization at the frequency range of 2–18 GHz is investigated. In the double-ridged horn antennas, the design is based on the starting frequency (e.g., the lowest working frequency). Figure 7a shows an example of a horizontally polarized horn antenna in the 3D software ANSYS HFSS with version number ANSYS HFSS 2022 R1, operating in the 2–18 GHz frequency band. Additionally, Fig. 7b presents the general view of the antenna implemented in SOLIDWORKS software with version number SOLIDWORKS 2023 SP2.0 (Service Pack 2.0).

**Fig. 8:**
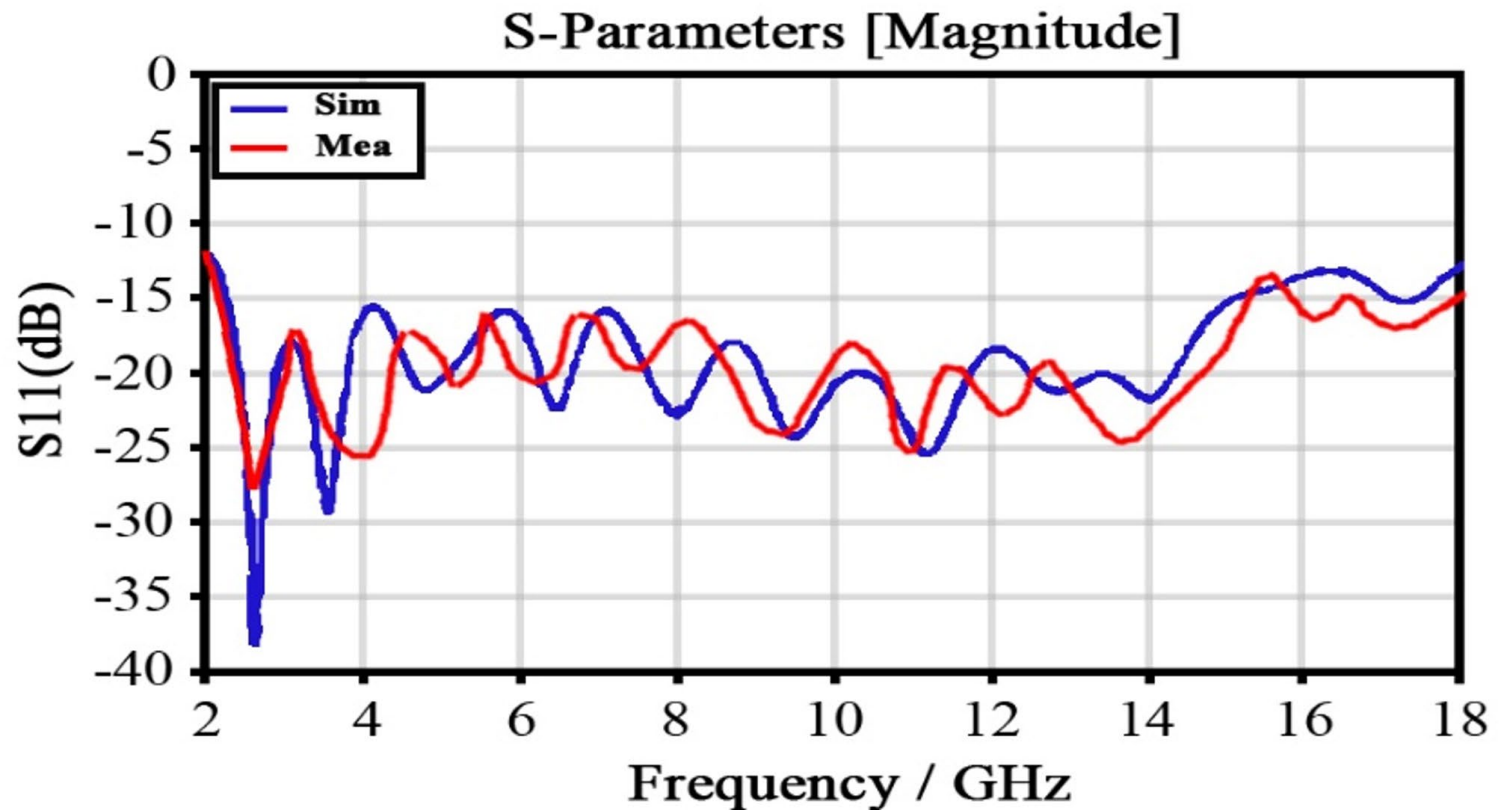
The diagram of the return loss double blade horn antenna with horizontal polarization.

**Fig. 9:**
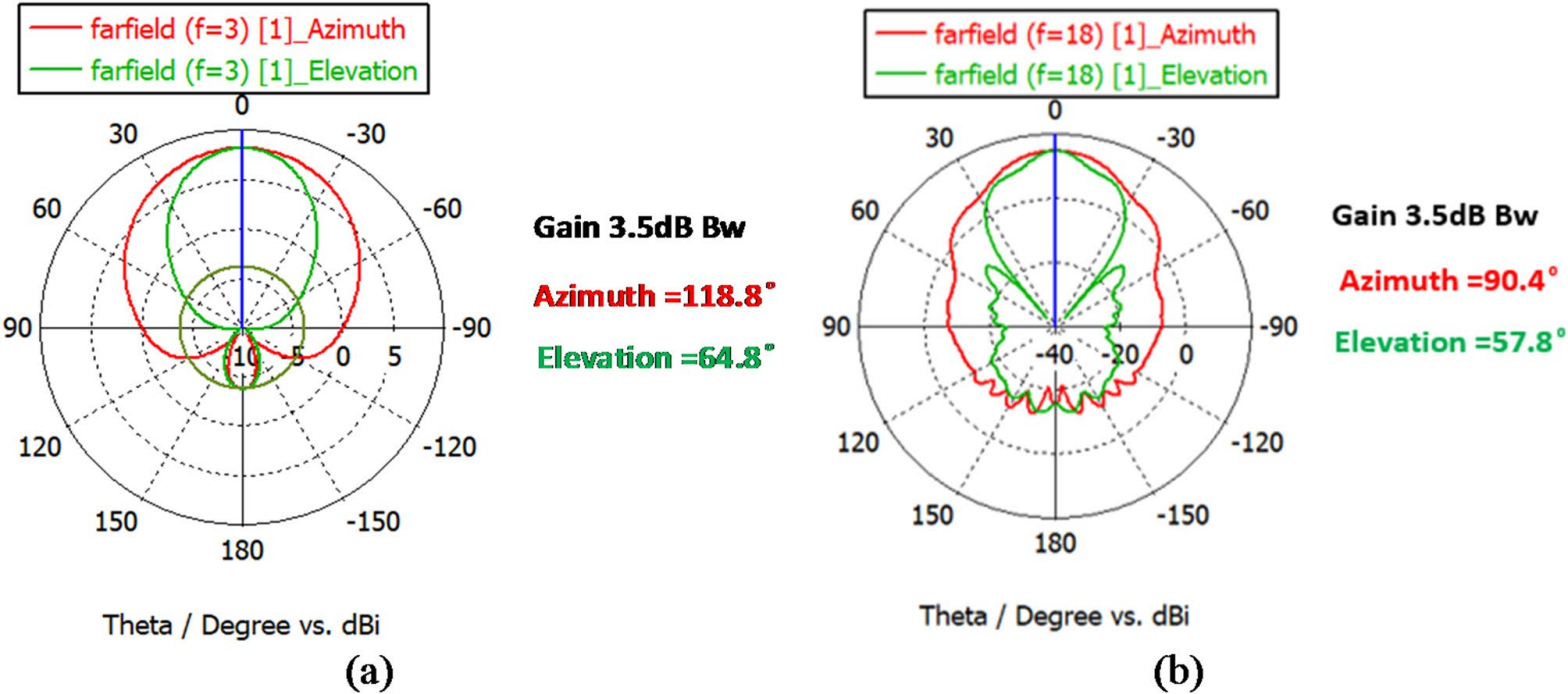
The radiation pattern of a two-blade horn antenna with horizontal polarization at **(a)** 2 GHz frequency and **(b)** 18 GHz frequency.

**Fig. 10:**
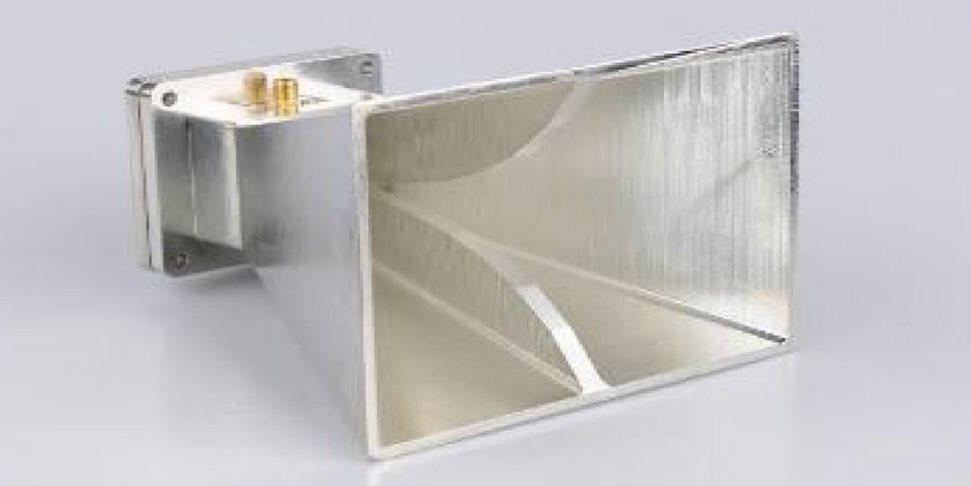
A fabricated antenna with horizontal polarization.

As seen in Fig. 8, the return loss coefficient in the horizontal antenna is – 10 dB in the entire 2–18 GHz frequency range. Figure 9 shows the radiation pattern at 2 and 18 GHz frequencies in plotted Azimuth and Elevation modes, which are indicative of above-90 degrees Azimuth and above-60 degrees Elevation. The main idea of the present study is the use of a proposed rectenna system in crewless combat aerial vehicles (UCAV) and drones. Generally, the results for various frequencies are presented in Fig. 10.

An important aspect of this research is the use of a new and innovative generation of ridge horn antennas to mitigate and reduce the disadvantages of traditional horn antennas, such as their weight and size. These ridge horn antennas not only offer broadband performance, covering a wide frequency range, but they are also much lighter, more compact, and more flexible compared to classic horn antennas.

**Fig. 11:**
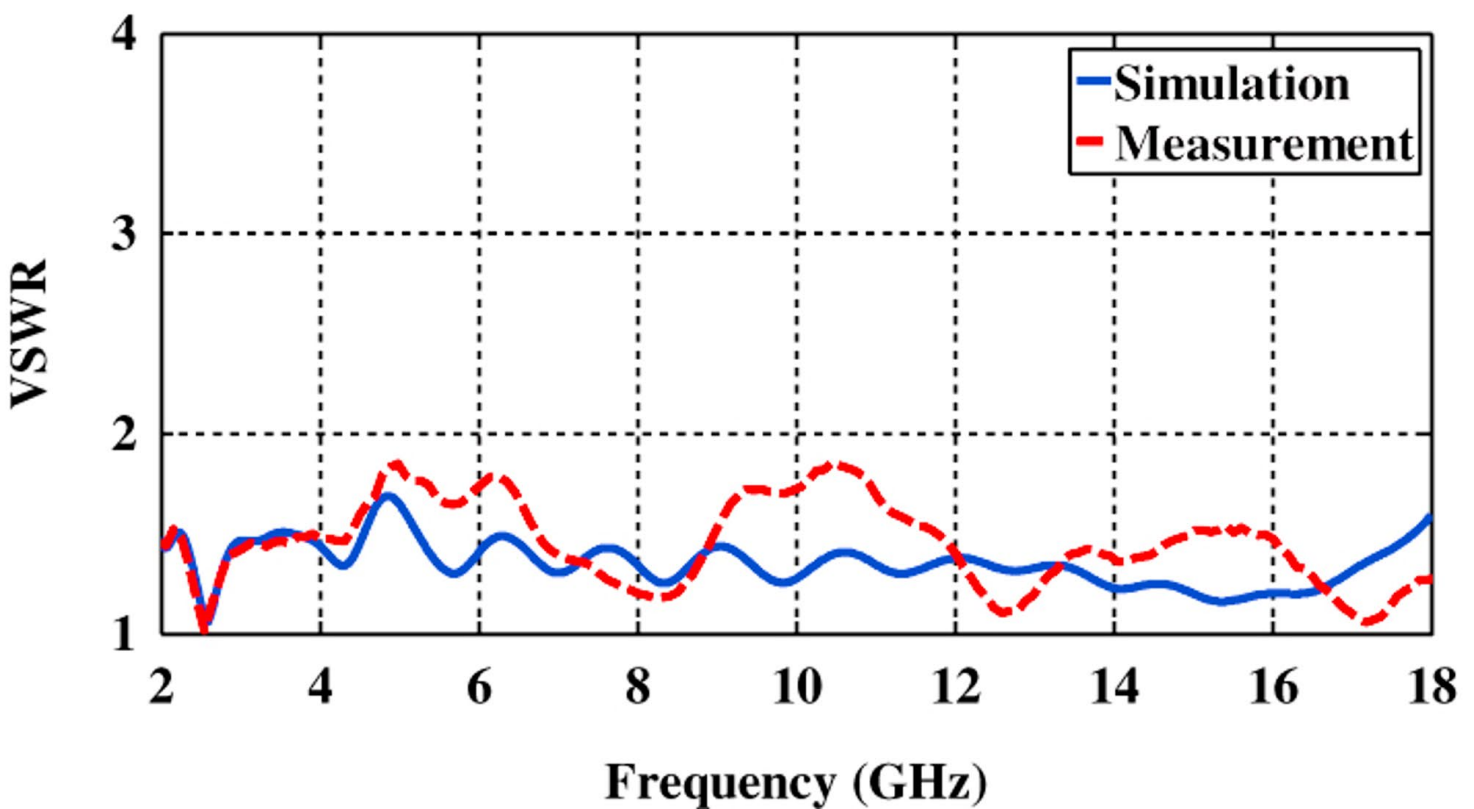
VSWR plot versus frequency for both the simulation and the fabricated broadband horn antenna.

As shown in Fig. 11, the VSWR is less than 1.7 across the entire frequency range, which indicates that the antenna is well-matched to the transmission line, with minimal reflection and efficient power transfer. A VSWR value lower than 1.6 suggests that the antenna maintains a good impedance match across the entire operating frequency range, meaning that the majority of the transmitted power is absorbed by the antenna rather than being reflected.

A VSWR of less than 1.6 across the entire frequency range indicates a well-matched antenna, with minimal reflection and efficient power transfer. This is beneficial for Rectana systems, as it enhances power efficiency, improves system performance, ensures effective multi-band or wideband operation, and facilitates seamless integration between the antenna and rectifier circuit. A lower VSWR also ensures better compatibility between the antenna and rectifier circuit, ensuring efficient and reliable operation.

**Fig. 12:**
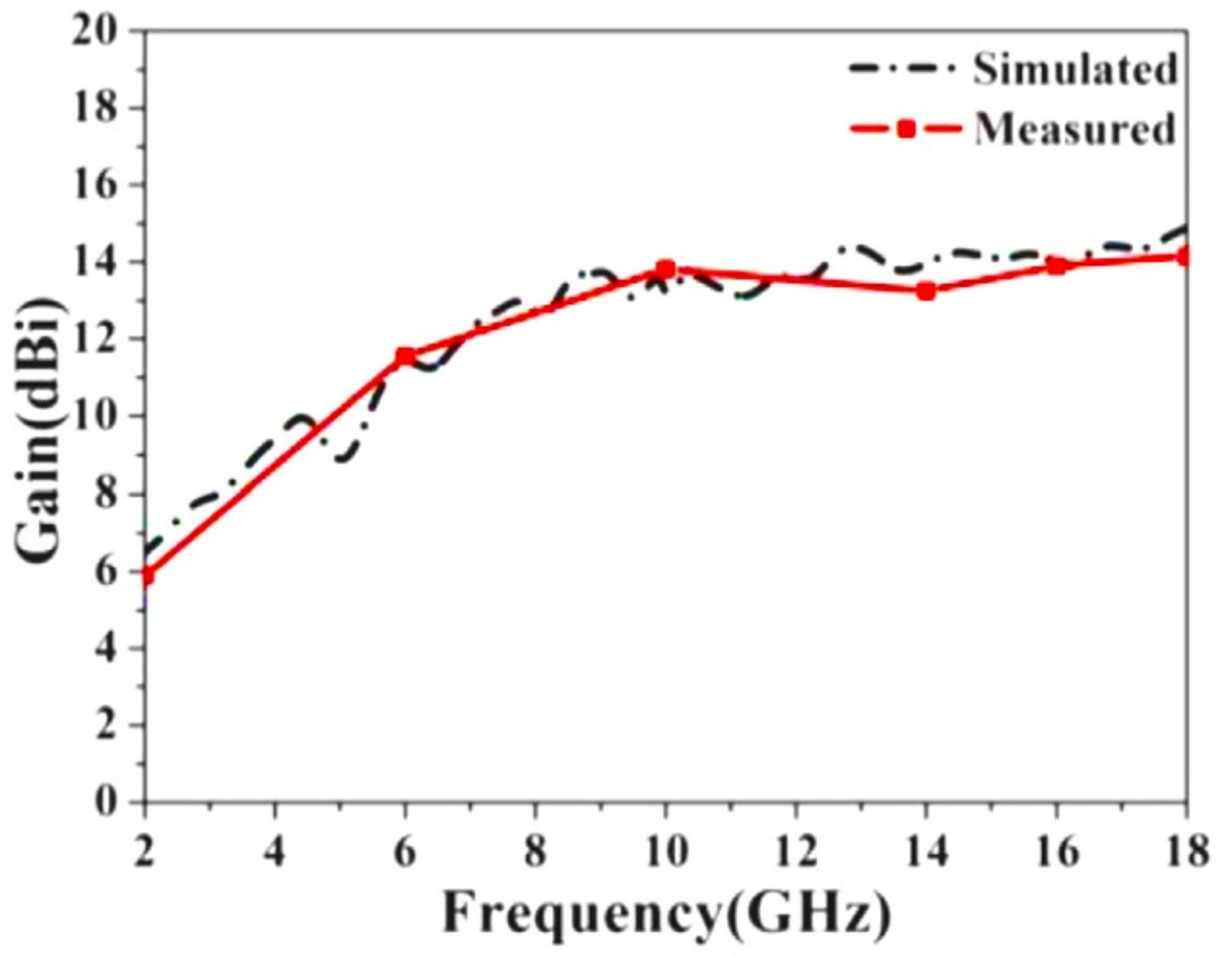
Gain plot versus frequency for both the simulation and the fabricated broadband horn antenna.

Figure 12 illustrates the gain of the horn antenna in both simulated and fabricated states within the 2–18 GHz frequency range. As shown in Fig. 12, there is excellent agreement between the simulation and fabrication results. High-gain, ridged horn antennas in rectenna systems improve signal reception and transmission efficiency by focusing energy in a narrower beam, enhancing directivity, boosting energy harvesting efficiency, allowing for longer range, and reducing interference. These characteristics make them useful in wireless power transfer, satellite communication, and high-frequency energy harvesting systems.

### Wilkinson power divider and combiner

**Fig. 13:**
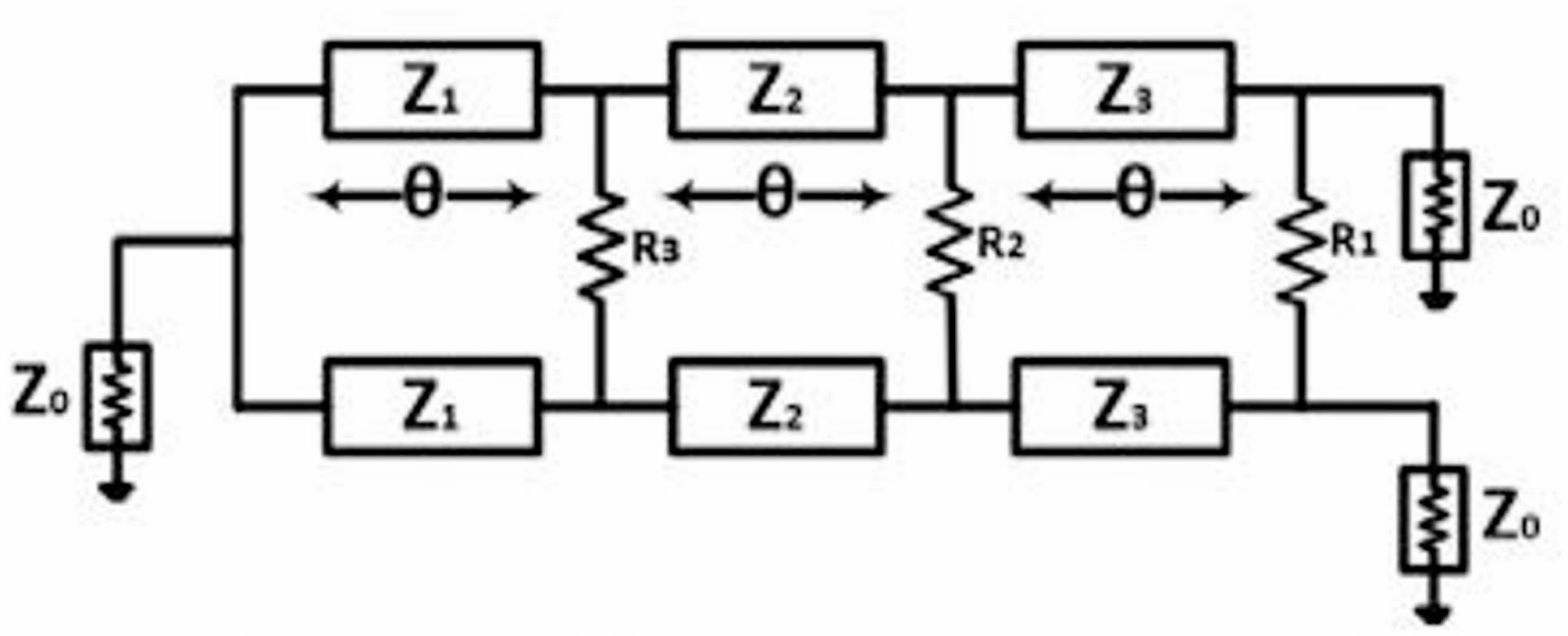
General schematic of multipart Wilkinson dividers.

The Wilkinson power divider is a circuit that can cause no specific problems or be very cost-effective. Impedance matching is done on the output branches. The output branches should be a quarter (λ/4) of the wavelength of the central frequency (f0). In addition, a width is chosen in a way that the special impedance of the line is the geometric center of input and output transmission lines. In the WPD in which the impedance matching is done on the output ports, the reflection coefficient drop is observed between the input and output. Moreover, the transmitted power in each branch can be increased up to – 3 dB. Generally, the classical WPDs are designed for a single frequency. The schematic of the multi-section WPD is shown in Fig. 13. All sections are equal in length (θ = λ/4). However, the thickness of the lines, which are indicative of the special impedances, is different. The input impedance in the entire broadband frequency range is equal to Z0 = 50 + j0. In this project, and for the design of the multi-section Wilkinson power dividers, the Chebyshev polynomials have been used to calculate the impedance of different sections and evaluate the effects of that section on other sections.

The main equation of the Chebyshev polynomials is presented in Eq. (1).

1 $${T_n}\left( x \right)=2 \times {T_{n - 1}}\left( x \right) - {T_{n - 2}}\left( x \right){\text{~}}$$

One of the special features of Chebyshev polynomials is that with the increase in the number of sections of a system, the fluctuations are also increased, although the bandwidth is increasing and the return loss and the reflections are decreasing. Modeling of the impedance matching in the Chebyshev polynomials is shown in Eq. (2).

2 $$\left( {{\text{sec}}{\theta _m}} \right)=\frac{1}{{{{\text{\varvec{\Gamma}}}_m}}}\left| {\frac{{{Z_L} - {Z_0}}}{{{Z_L}+{Z_0}}}} \right| \approx \frac{1}{{2{{\text{\varvec{\Gamma}}}_m}}}\left| {\ln \frac{{{Z_L}}}{{{Z_0}}}} \right|~$$

In Eq. (2), in which θm is the calculated angle for a bearable and acceptable reflection coefficient (Γm), ZL is indicative of the load impedance, and Z0 is indicative of the special impedance of the transmission line. Generally, the Chebyshev polynomials of the total reflection are shown in Eq. (3), in which the TN (secθ) is calculated by matching the Chebyshev polynomial degree of n. In Eq. (3), Γ is the reflection coefficient and θ is the angle of the desired line per wavelength. The line angle is the angle corresponding to the quarter wavelength, and A is the design factor. The formula proposed in Eq. (4) is a parameter calculated separately for each section.

 

3 $${\text{\varvec{\Gamma}}}\left( \theta \right)=A \cdot {e^{ - jN\theta }}{T_N}\left( {\sec {\theta _m}\cos \theta } \right)~~$$

4 $$A=\frac{{{Z_n} - {Z_{n - 1}}}}{{{Z_n}+{Z_{n - 1}}}}\frac{1}{{{T_N}\left( {sec{\theta _m}} \right)}}~$$

The real impedance matching of multi-section circuits, which theoretically have n parts with equal lengths (L), is equal to a quarter of the wavelength at impedance matching sections. However, to make the Wilkinson divider broadband, regular loops have been used. Inter-loop resistance is calculated by analysis of even and odd modes in the calculation of even mode.

5 $$E_{n}^{N} \left( x \right) = \mathop \sum \limits_{{n = 0}}^{N} \left( {\begin{array}{*{20}l} N \hfill \\ n \hfill \\ \end{array} } \right)\frac{{E_{n} }}{{2^{n} }}\left( {x - \frac{1}{2}} \right)^{{N - n}} ~$$

**Fig. 14:**
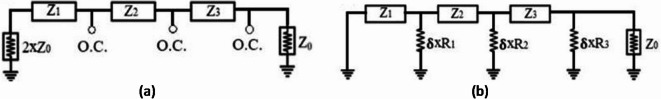
A schematic of the multi-section Wilkinson power divider circuit (a) Odd mode and (b) even mode.

Each of these resistors between the sections is converted into an open circuit (OC). Because there are equal divisions for the two separate outputs, the special impedance of the source that is placed on our circuit’s input is equal to 2 x Z0. All the inter-loop resistances are calculated using the Euler Eq. (5) in the even and odd modes. In the above Eq. (5), N is the highest degree of the Euler function. To be precise, Fig. 14 can be divided into the odd and even sections, as shown in Fig. 14. When moving from one section to another, the Rn resistors, one end of which is short-circuited, are placed between the loops.

**Fig. 15:**
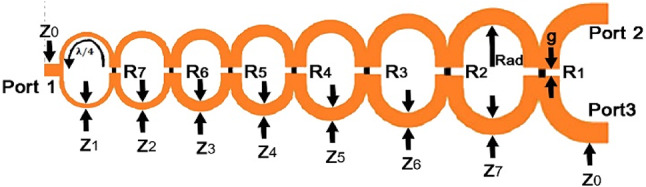
Schematic of wideband Wilkinson divider in high-frequency simulator software.

Regarding the abovementioned analyses and equations, the broadband Wilkinson power divider is designed and simulated in the CST software, as shown in Fig. 15. Also, the load impedance was calculated at the end of each output section. Using the mathematical analysis and the calculations in the Euler and Chebyshev equations, we have Z1 = 87.8 Ω, Z2 = 67.9 Ω, and Z3 = 55.5 Ω. In this design, a Rogers 4003c substrate was used as a cheap substrate with a dielectric coefficient of 3.38 and a loss tangent coefficient of 0.0027 in the power divider substrate.

**Fig. 16:**
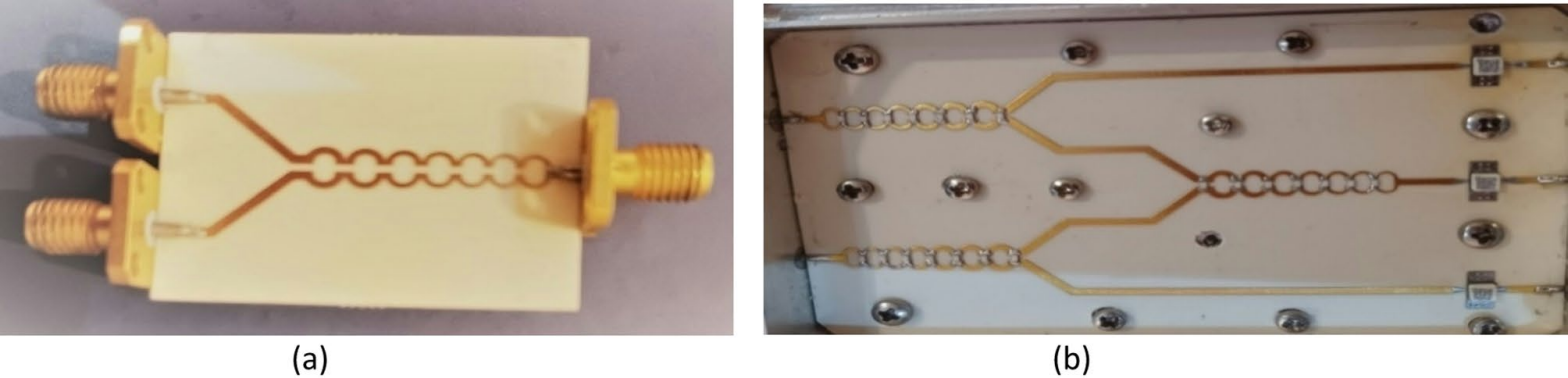
The fabricated wideband Wilkinson power divider (a) single and (b) the wideband Wilkinson combiner and power divider with different polarizations.

Each section is dependent on the previous one, which causes bandwidth defects in this matching technique. However, in the present technique, this defect is prevented by the use of regular and close cascades. Also, through the creation of 7 loops, it makes fluctuations in the 2–18 GHz frequency range. A prototype of a single-element Wilkinson power divider is fabricated as shown in Fig. 16a.

Due to the use of two antennas with horizontal and vertical polarizations, a Wilkinson power divider formation is needed in which the polarizations can receive and manage signals in all directions, both separately and in combination. In addition to rectenna systems, this power divider system can also be used in all low cross-section micro air vehicles (MAVs) as an automated and renewable power source. To do so, a formation (Fig. 16b) of these signals is presented with a regulator and stabilizer at the end of its structure to provide the desired frequency band. In the design of the Wilkinson power divider, which divides the power into two equal sections, Zo is the special impedance, which is equal to ohm, and the impedance of every other part is also calculated from the third term of the Chebyshev polynomial.

### Rectifier design

The rectifier section in a system is crucial, and the Schottky diode is a key component used for rectification. It is characterized by low threshold voltage, high switching speed, and minimal losses. The proposed work operates in a wideband frequency range of 2–18 GHz with low power absorption, so further investigation into the selection of this particular diode is necessary. The Schottky diode used in this study is the HSM285O, which was incorrectly referred to as HSM-285 C in the text.

Schottky diodes are commonly used in high-frequency applications such as rectennas due to their low forward voltage drop, fast switching speeds, and high-frequency performance. The diodes should be evaluated based on low forward voltage drop, fast switching speed, low series resistance, high reverse breakdown voltage, and low junction capacitance.

Comparing diodes for 2–18 GHz rectenna applications, the HSMS-285O diode is selected due to its low capacitance, low forward voltage drops, fast switching speed, and high-frequency operation. The HSMS-285O diode is suitable for rectenna systems in the higher-frequency range, especially in the middle portion of the 2–18 GHz frequency range.

**Fig. 17:**
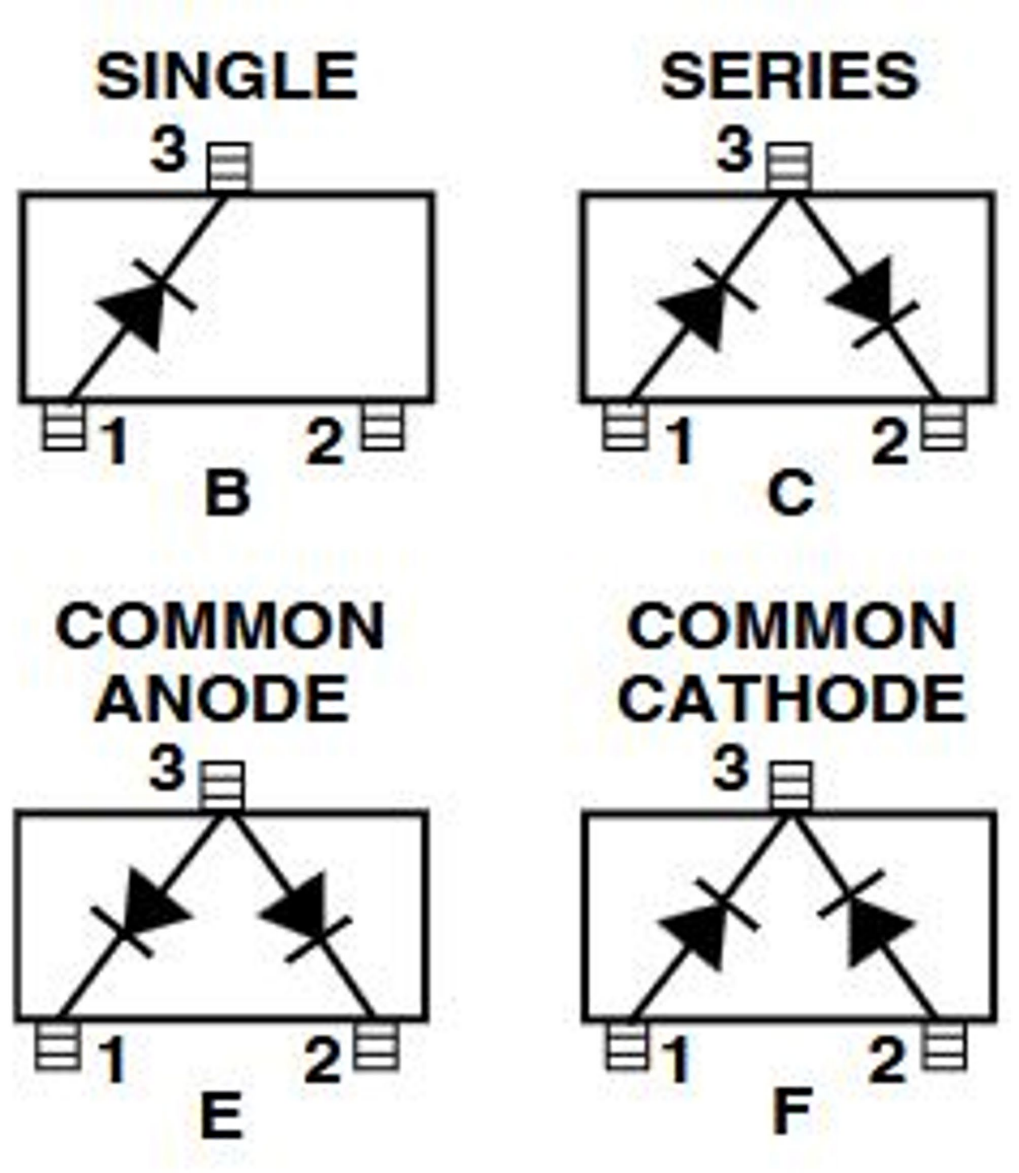
The packaging schematic and placement of the Schottky diode HSMS-285O in various configurations.

The various schematics of the HSMS-2850 Schottky diode in different packaging configurations are shown in Fig. 17.

**Fig. 18:**
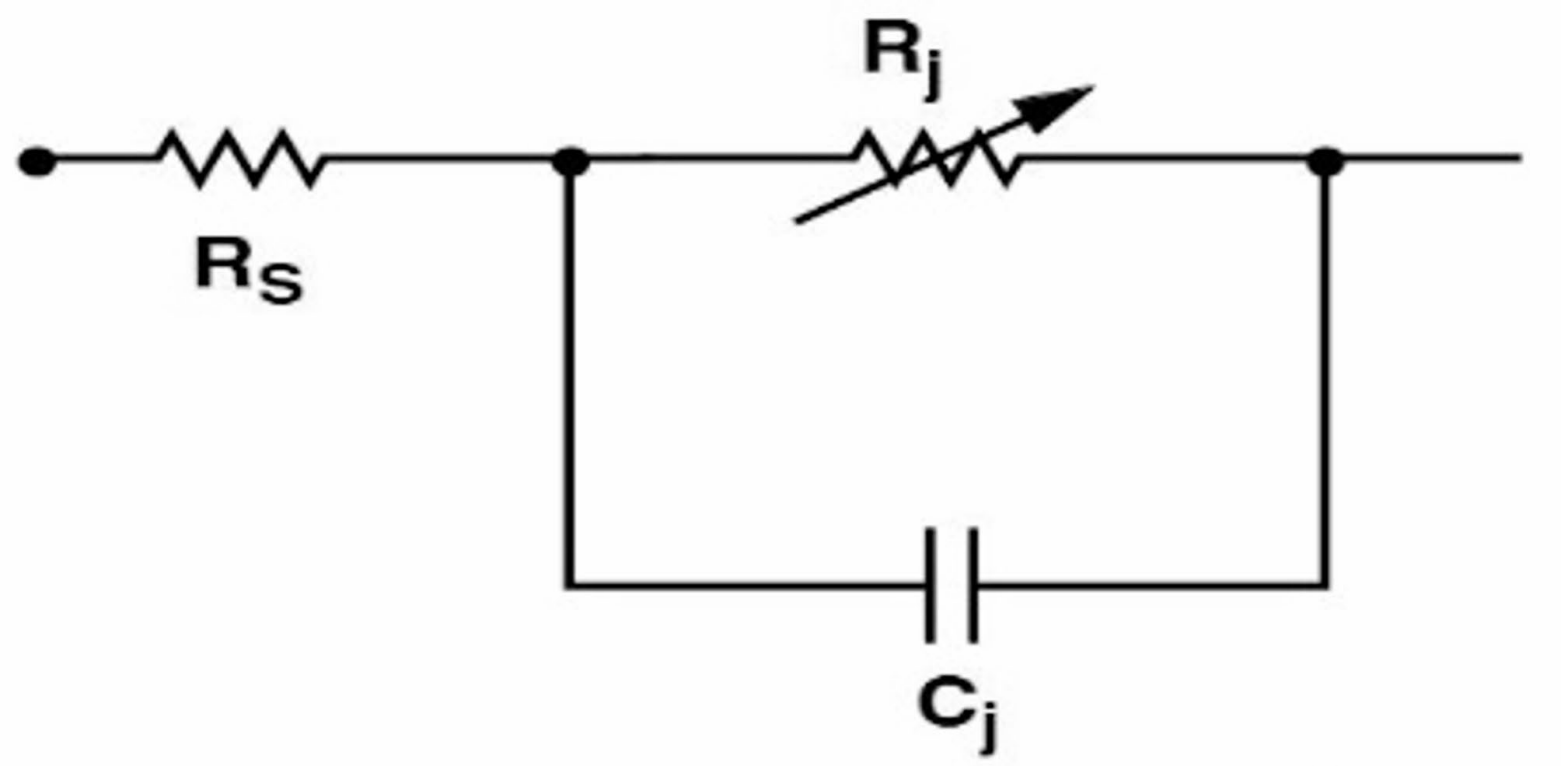
The linear circuit model of the Schottky diode in the proposed rectifier.

Considering the content of the research, which involves not only a wideband range but also addresses DC voltage, this factor led to the selection of the Schottky diode in a common anode series (E Sample) configuration. The linear circuit model of the HSMS-2850 Schottky diode is shown in Fig. 18.

In the above equivalent circuit, RS is the series resistance, Cj is the junction capacitance, and Rj is the variable junction resistance.

Avago’s HSMS-285x family of Schottky detector diodes has been developed specifically for low cost, high.

volume designs in small signal (Pin < -20 dBm) applications at frequencies below 1.5 GHz. At higher frequencies, the DC-biased HSMS-286x family should be considered.

In large signal power or gain control applications (Pin > -20 dBm), the HSMS-282x and HSMS-286x products should be used. The HSMS-285x zero-bias diode is not designed for large signal designs.

**Fig. 19:**
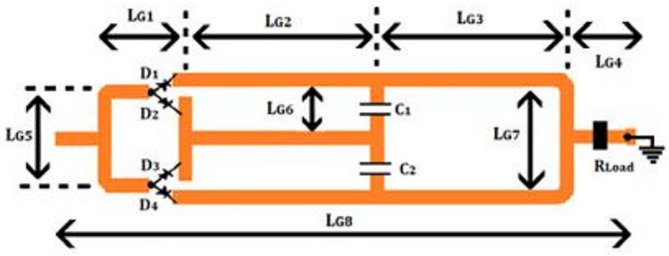
Proposed rectifier circuit of RF energy harvesting.

The RF4 substrate has specifications similar to those of materials used in the Wilkinson divider on the rectifier. Here, an HSMS-285 C Schottky diode is chosen with specific parameters such as RS = 25 Ω as the series resistance, Cj = 0.18 pF as the short circuit capacitor capacity, Ib = 3 × 10 –6 A as the bias current, and Is = 3 × 10 –6 A as the saturated bias. Also, Is = 3 × 10 –4 A is provided in this Schottky diode equivalent circuit. Each of the four diodes used provides a DC voltage of 2.2 V on a load with rectification of the entire wave. The capacitors with values of C1 = C2 = 100pF prevent fluctuations in the rectification of the entire wave. The thickness of the transmission line is 1.5 mm in the entire rectifier circuit. The length of the rectifier circuit is LG1 = 2.3, LG2 = 11.9, LG3 = 12.9, LG4 = 2.6, LG5 = 5.7, LG6 = 3.9, LG7 = 9.2, and LG8 = 35 mm. To obtain the output voltage specifically, the impedance is considered to be 1500 Ω. According to the explanations and the design, the schematic of the rectifier is presented in Fig. 19. It should be noted that by creating a hole in the Roger substrate, the load resistors are connected to the ground.

**Fig. 20:**
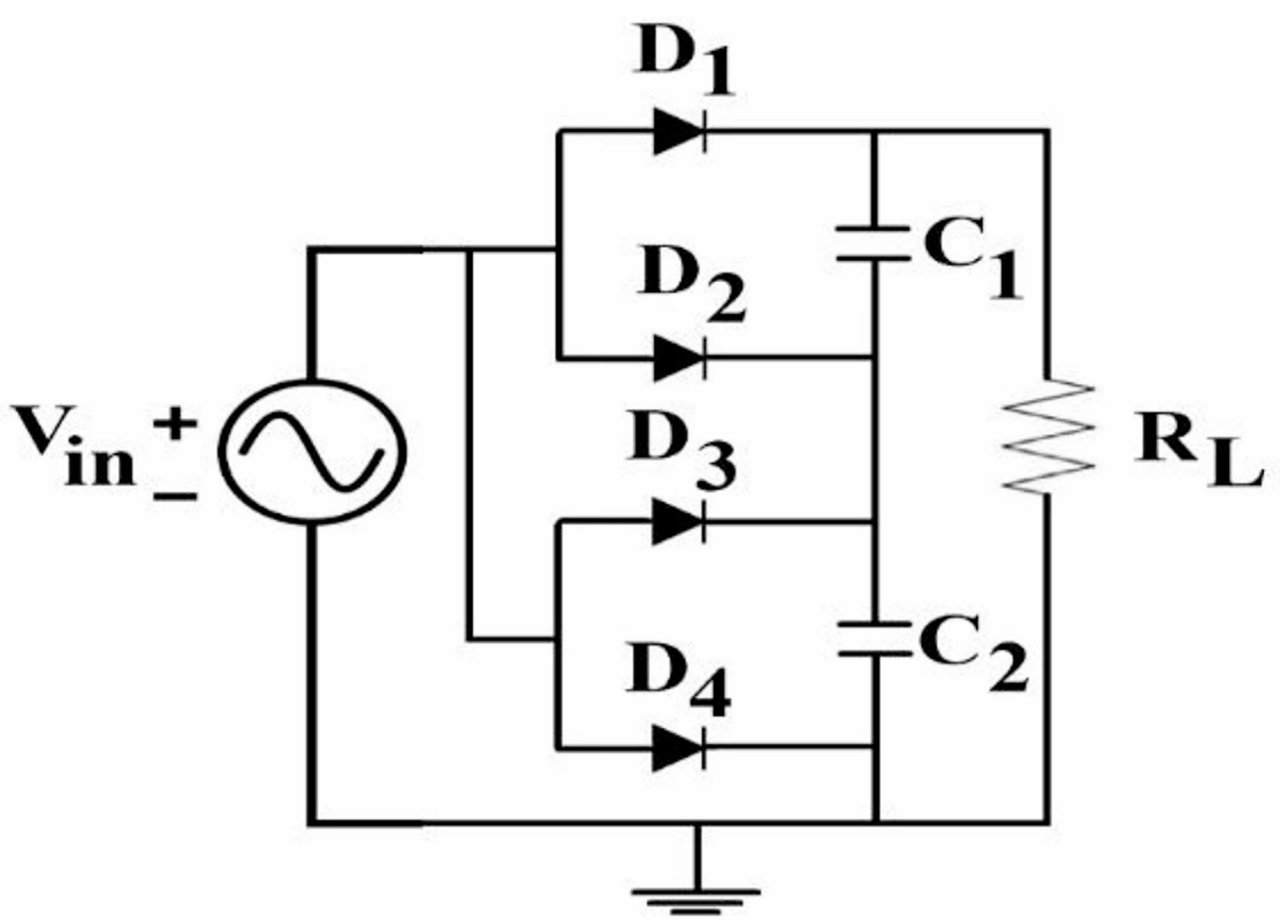
The circuit section of the proposed rectifier, including all components used, such as diodes and capacitors.

In general, the circuit structure of the upper and lower sections each consists of two Schottky diodes and one capacitor (resulting in a total of four Schottky diodes and two capacitors). The diodes ensure that both half-cycles of the alternating signal (Vin) are converted to positive, while the capacitors filter out the fluctuations in the positive signal. In the optimal configuration, a similar structure is designed integrally. As a result, a suitable direct voltage (V out) can be obtained across the output resistor. Below the FR4 substrate, the dielectric constant of the substrate is 4.3, and the loss tangent is 0.025. Generally, the circuit configuration of the rectifier is shown in Fig. 2, where the characteristic impedance of the transmission.

line is 50 ohms. Figure 20 shows the equivalent circuit of the proposed rectifier circuit in the ADS software.

The equivalent resistance value is 1 kΩ, which represents the optimal condition for the rectifier, as determined based on simulation results with various values.

**Fig. 21:**
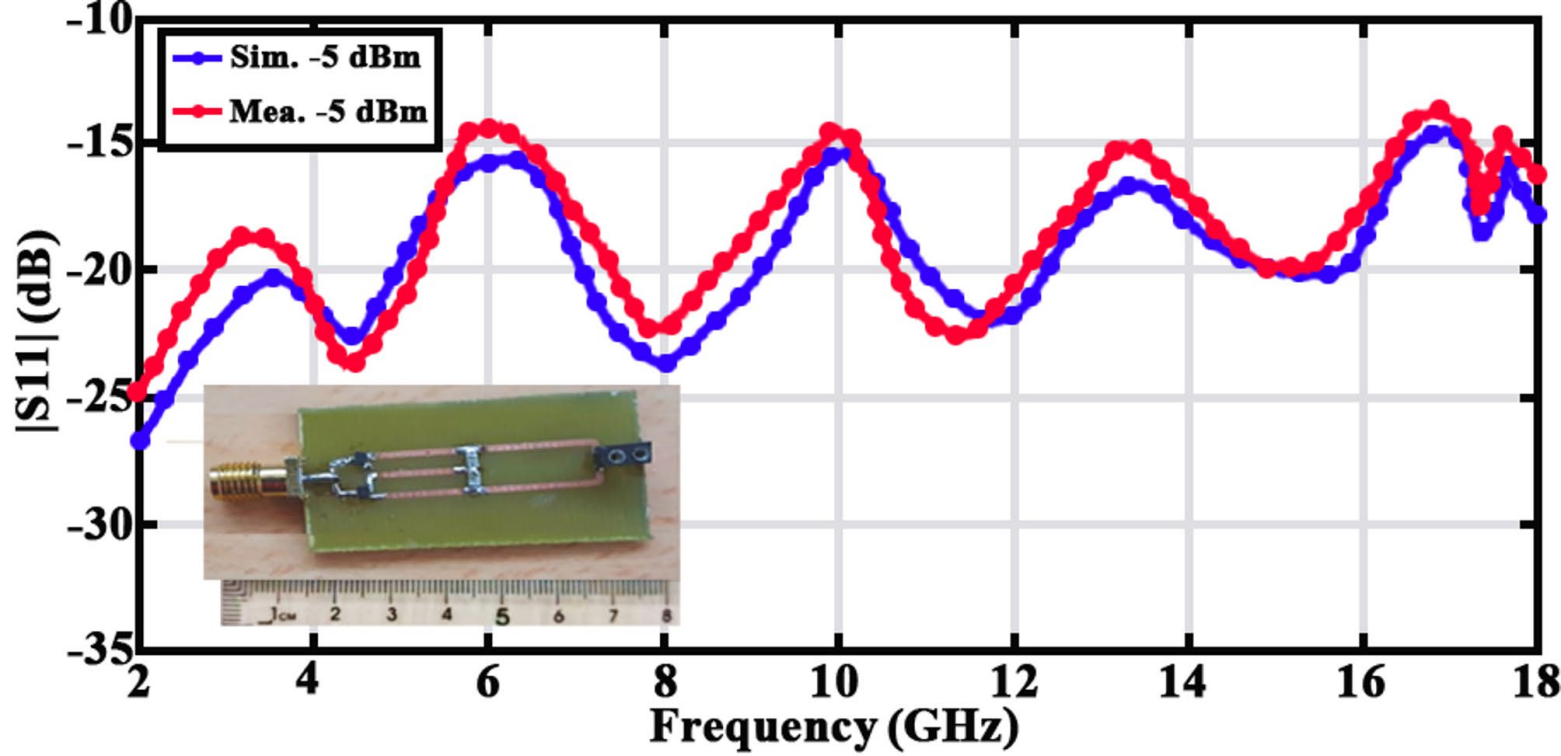
Simulation and measurement of |S11| for a stand-alone rectifier at an input power of -5 dB, using a 1kΩ load resistor.

To analyze the performance of the rectifier section, the return loss versus frequency chart should be examined over the entire range of 2–18 GHz. According to Fig. 21, the return loss curve remains below minus ten decibels throughout the entire frequency range. This indicates that the rectifier exhibits very good matching with other sections of the rectenna system.

As presented in most of the studies on the rectifier antenna, the conversion efficiency is very important. The value of this efficiency, which is equal to the ratio of output power (the direct power generated on the output) to the input power, is calculated by the following Eq. (6).

6 $$~{\text{PCE}}\left( {\text{\% }} \right)=\frac{{{P_{DC}}}}{{{P_{in}}}} \times 100{\text{\% }}=\frac{{{V^2}DC}}{{{R_L}{P_{in}}}} \times 100{\text{\% ~~~}}$$

**Fig. 22:**
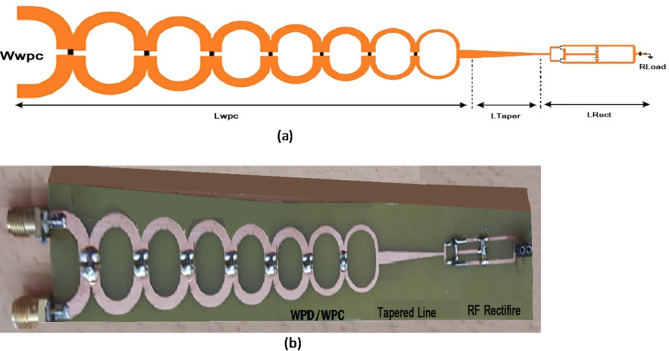
The position of the proposed rectifier and divider circuit **a** Schematic image in the software; **b** Fabricated integrated circuit.

The Power Conversion Efficiency (PCE) has been investigated for the frequency range of 2–18 GHz. It is 55% for the lower input powers, such as -15 P_in_, and with the increase in the input power, it is increased to 65%. The proposed design can first harvest the different modes and positions of ambient radio energy. Also, it can provide a cheap substrate for a frequency range in the form of various polarizations. Figure 22 presents a schematic of an integrated Wilkinson divider and combiner can be seen, there is no matching between the impedances of these two lines. Therefore, to prevent this defect, a linearly changing taper from 2.9 mm (Z_0_ = 2.9 mm) to 1.5 mm is added between these two sections with a length of 18/85. With the addition of this line between the two sections, the triple-port divider is divided into two equal sections. With these interpretations, the length of the Wilkinson divider is 105 mm, the size of the impedance matching line between the two parts is 18.85 mm, and the size of the rectifier is 36 mm.

**Fig. 23:**
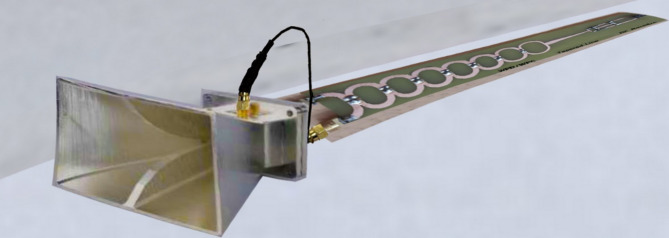
Actual image of the fabricated Rectana prototype, including the horn antenna, power divider, and integrated rectifier.

In alignment with your valuable comment, a schematic overview of the rectenna receiving system, including the Receiving Antenna, the rectifier, and the broadband Wilkinson power divider, is presented in Fig. 23.

**Fig. 24:**
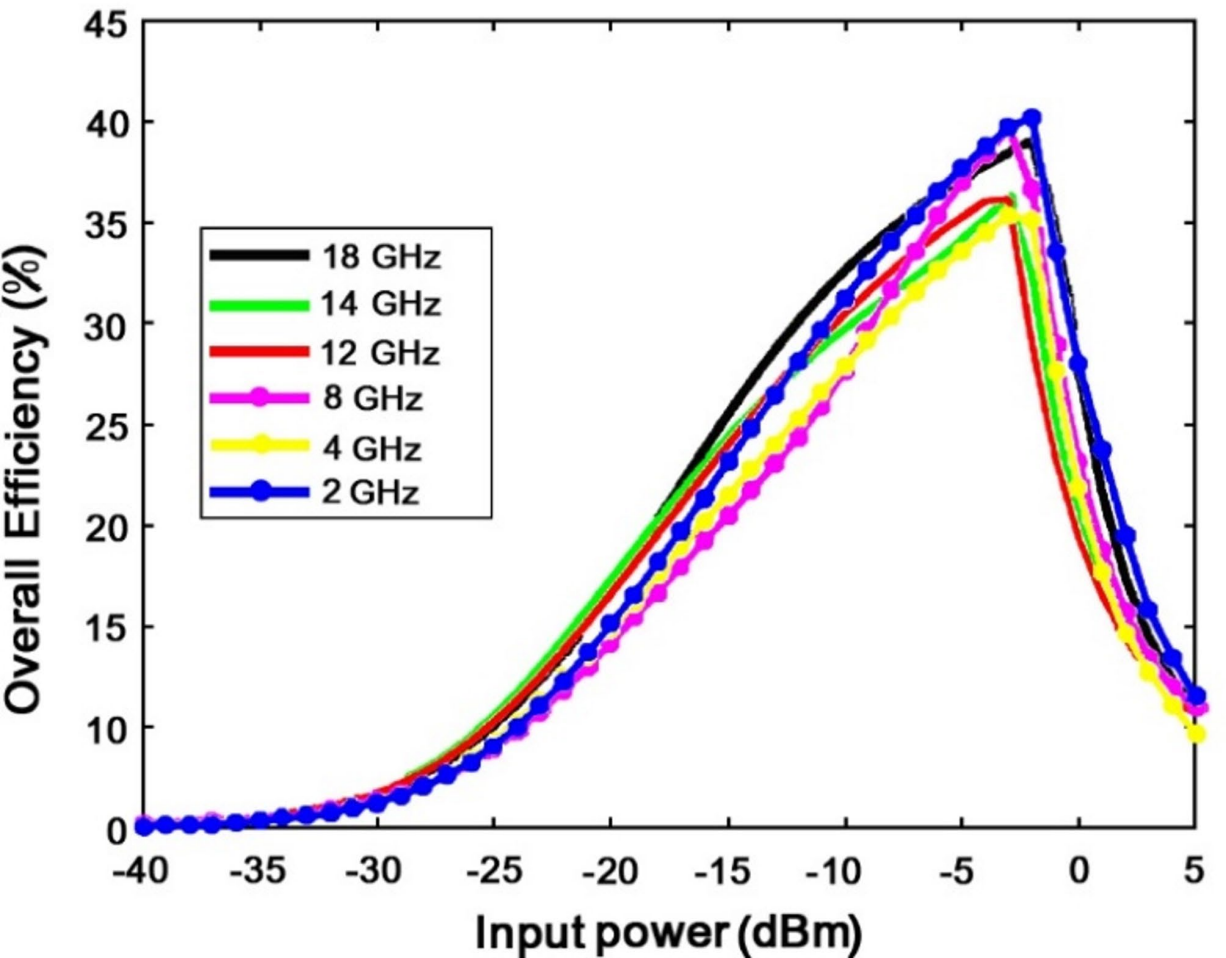
Diagram of power conversion efficiency of the proposed rectifier circuit at different frequencies.

Figure 24 shows the graph of transformation efficiency per input power. As seen in Fig. 24, in power values lower than − 5 also, the power conversion efficiency will be 20%.

**Fig. 25:**
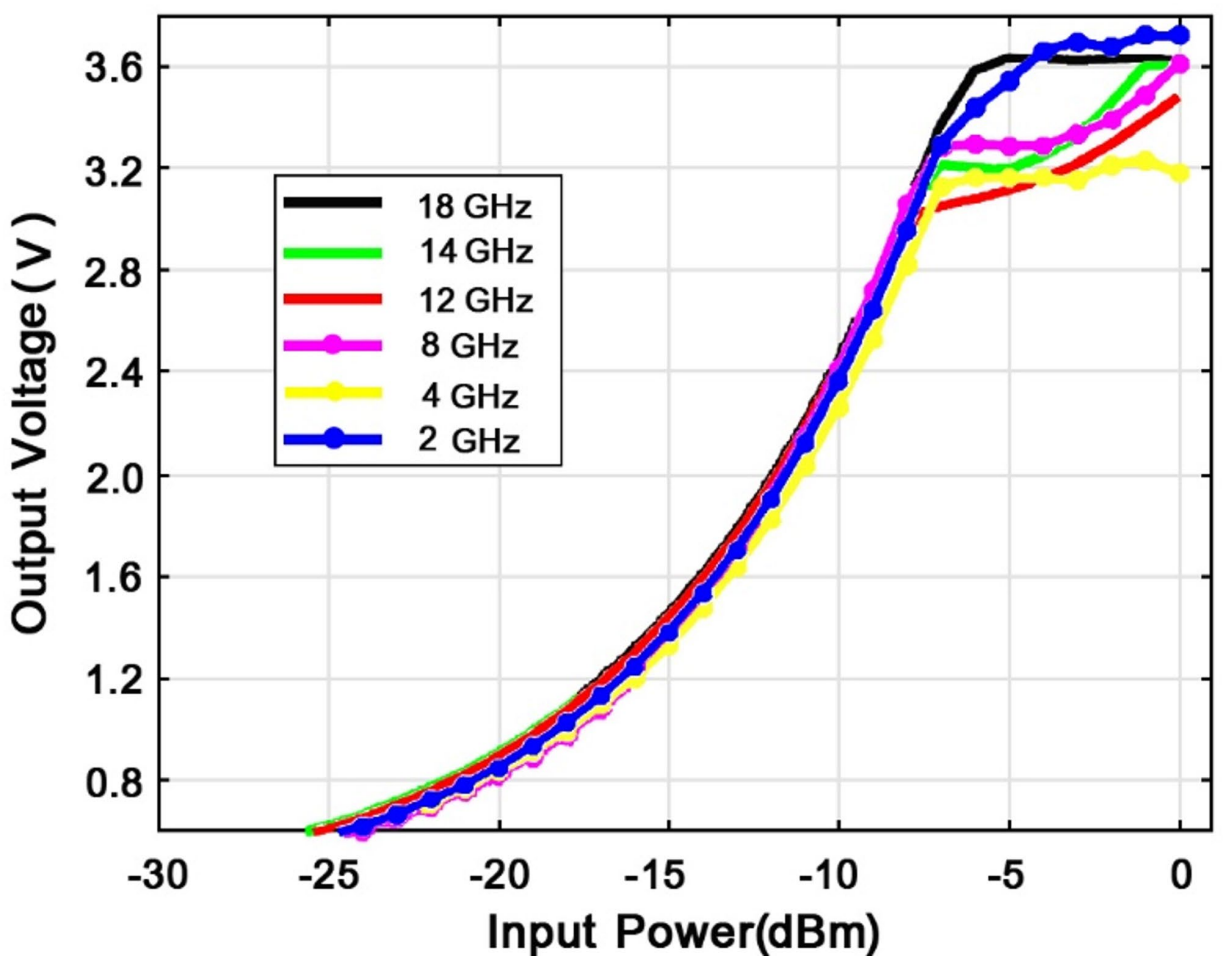
Output voltage diagram according to input power in different frequency bands.

Figure 25 shows the proposed Rectenna output voltage as a function of input power for different operating frequencies. As seen in the figure, for an input power of -5 dB, the output voltage exceeds 3.55 volts across all operating frequencies. It is worth noting that the maximum output voltage of 3.65 volts occurs at a frequency of 2 GHz.

**Fig. 26:**
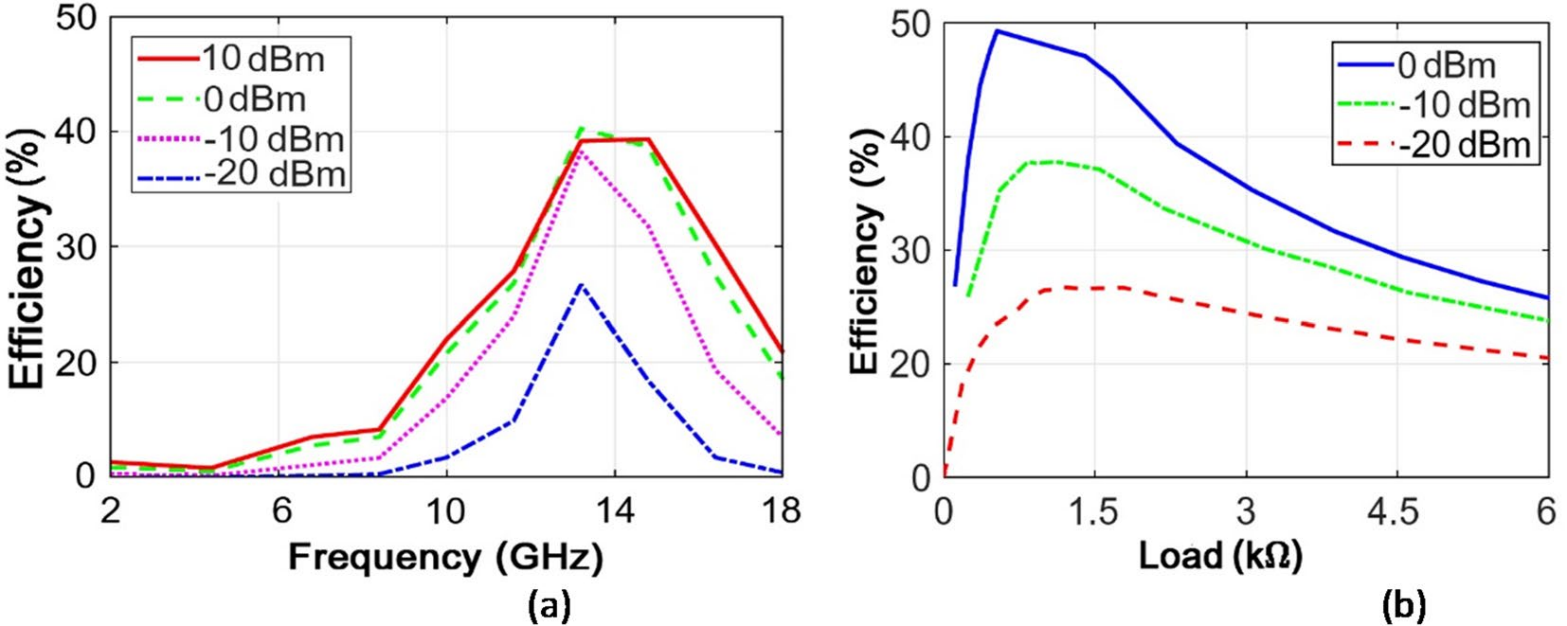
Power conversion efficiency diagrams in terms of (**a)** frequency and (**b)** load resistance.

As seen, according to the processes in all rectenna systems, with the decrease in the input power, the power efficiency is also decreased. However, what should be noted about the proposed system in Fig. 26b is that with the decrease in the input power, the conversion efficiency in the very low powers (under − 20 dBm) is still above 25%. Figure 26a illustrates the power conversion efficiency as a function of frequency for input power levels ranging from − 20 dB to + 10 dB. As shown in Fig. 26a, within the frequency range of 10 to 18 GHz, the power conversion efficiency exceeds 20% for an input power of 0 dBm. This indicates that the proposed system demonstrates efficiency and performance across a wide range of lower input power levels. Figure 26b shows the power conversion efficiency as a function of load resistance for input power levels ranging from − 20 dB to 0 dB. According to Fig. 26b, the highest power conversion efficiency is achieved at a load resistance of 1 kΩ across various input power levels.

**Fig. 27:**
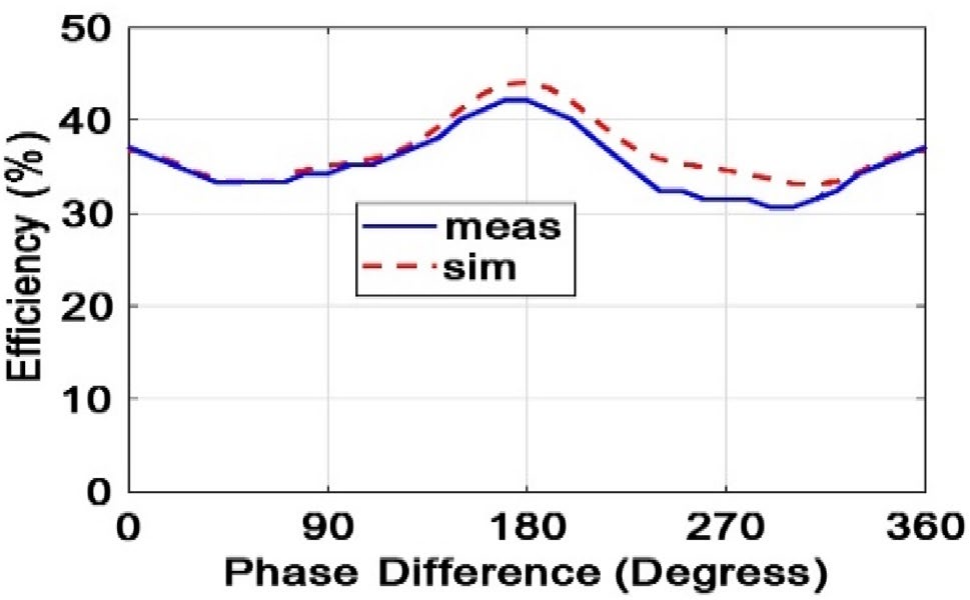
Graph of power transformation efficiency per phase in the simulation mode and the measurement of the integrated rectenna.

As seen in Fig. 27, when input signals are in the phased mode, the transformation efficiency is maximized and optimized. In addition, it is zero when the phase difference is rewound 180 degrees or 360 degrees in a regular period. Figure 16 shows that the power conversion efficiency in the input power is less than − 5 dB in all working frequency ranges, more than 32%. Figure 18 shows that the proposed rectenna system has performance and efficiency at very low input power (input power less than − 20 dBm).

Two signals with different phases and an input power of − 5 dB are applied to the rectifier circuit shown in Fig. 22b. One of the signal generators varies from 0 to 360 degrees, while the other remains fixed at a phase of 0 degrees. According to Fig. 27, with a phase change from 0 to 360 degrees, the power conversion efficiency (efficiency) varies between 32% and 40%, indicating that the proposed rectifier performs consistently across different phases.

The advantages of phase alignment and covering a wide phase range for the rectenna system include improved energy harvesting efficiency and greater robustness. Phase alignment ensures optimal power transfer when the input wave is in phase with the rectenna components, leading to maximum energy conversion. A wide phase range ensures that the rectenna system can effectively harvest energy from signals with varying phase shifts, enhancing its flexibility and performance under different operational conditions, such as varying signal sources or interference scenarios. This adaptability increases the overall reliability and efficiency of the rectenna system in real-world applications.

As seen in Table 1, the transformation efficiency is 20% on average in the presence of a broadband frequency range. Also, the voltage is about 10% higher based on other authenticated references. In addition, the ambient radio wave harvesters can also work in very low powers. Regarding the simultaneous use of two antennas, this system can also receive waves from various angles. Another innovation is the separation and division of the polarization combination on the output of the divider circuit.

**Table 1:** Comparison between the important parameters of the rectenna system with other authoritative sources.

References	Frequency (GHz)	DC voltage (V)	Rectifying element	BW (GHz) @ ᾓ (> x%) at Pin (dBm)	ᾓ _max_ @ *P*_in_ at Frequency (GHz)	Pin range (x to y dBm) @ ᾓ > z(%)
^9^	5.8	1.8	BM870630	5.1–5.8@ >50% at -5 dBm	78%*** @ 0 dBm at 5.8	20dBm (-10to + 10) @ >550%
^10^	0.924–2.45	0.2	HSMS-285 C	1.4–2.6@ >30% at 0 dBm	60%*** @ 10 dBm at 1.8	18dBm (-1to + 17) @ >30%
^12^	29–46	0.8	MA4E1317	29–46@ >40% at + 5 dBm	80%*** @ 5 dBm at 32	50dBm (-20 to + 30) @ >27%
^14^	1–3	0.9	HSMS2852	1.5–3@ >28% at 10dBm	40%*** @ 5 dBm at 2.45	15dBm (-5 to + + 10) @ >25%
^16^	2.45	0.3	HSMS-2818	2–2.5@ >20% at 17dBm	40%*** @ 17.5 dBm at 2.4	1.5dBm (17 to + 18.5) @ >40%
^17^	3-6.5	1.2	HSMS 2862	1.8–2.2@ >30% at 10 dBm	45%*** @ 15 dBm at 4.4	10dBm (0 to + 10) @ >30%
^24^	1.6–2.4	1.6	HSMS − 2860	1.8–2.2@ >40% at 10 dBm	50%*** @ 5 dBm at 2	NA
^25^	1.8–3.5	0.15	SMS7630	1.8–2.50@ >30% at -5 dBm	55%*** @ 0 dBm at 1.85	15dBm (-1 to + 5) @ >30%
^28^	1.6–3.8	1.1	HSMS-282E	2.1–2.45@ >60% at -5 dBm	68%*** @ -5dBm at 2.45	20 dBm (-12to 0) @ >35%
^36^	0.4-1	0.7	SMS7630 in VD	0.4–0.8@ >20% at 20 dBm	36%*** @ 40dBm at 0.49	20 dBm (40to 60) @ >25%
^40^	1.6–2.8	0.8	HSMS2850	1.6–2.8@ >55% at 0 dBm	65%*** @ 0 dBm at 2.1	15 dBm (-10to + 5) @ >35%
^52^	1.5–3.5	1.5	SMS7630-040LF	2.1–2.7 @ >15% at 3 dBm	30%*** @ 8 dBm at 2.45	18 dBm (-18 to 0) @ >20%
^54^	2.25–3	2.1	HSMS2860 in shunt	2.25–3 @ >60% at 13 dBm	80.4%*** @ 13 dBm at 2.35	15 dBm (0 to 15) @ >55%
^57^	0.9	NA	HSMS2852	0.45–0.9 @ >50% at 0 dBm	66%*** @ 0.9 dBm at 2.350.9	16 dBm (− 1 to 15) @ >50%
Proposed	2–18	3.6	HSMS-2850	10–18 @ >20% at 0 dBm	40%*** @ 0 dBm at 2	17 dBm (− 17 to 0) @ >23%

NA: Not Available.

## Conclusion

In the proposed design, first, a broadband rectifier was offered using a Wilkinson power divider at frequencies of 2–18 GHz. All the substrates of this rectenna system are analyzed, designed, and fabricated separately. After validating each section in this rectenna system, an integrated rectifier circuit that can receive the horizontal and vertical polarizations is modeled to receive all the ambient radio waves. In the present study, a rectenna system that usually includes the single- and multi-frequency modes of a broadband frequency that works in X and KU bands has been designed and fabricated. Moreover, based on the results obtained, it can be seen that the proposed system has an ideal mode in terms of rectenna parameters such as the power transformation efficiency, high DC voltage and current, and functionality in very low powers. Also, the power conversion efficiency at low input power (less than − 20 dBm) is 30% on average.

## Data Availability

All data generated or analyzed during this study are included in this published article.
